# The effect of plasma from septic ICU patients on healthy rat muscle mitochondria

**DOI:** 10.1186/s40635-016-0093-2

**Published:** 2016-07-07

**Authors:** Jonathan Grip, Towe Jakobsson, Nicolas Tardif, Olav Rooyackers

**Affiliations:** Department of Anesthesiology and Intensive Care, Clintec, Karolinska Institutet and Karolinska University Hospital, Stockholm, Sweden

**Keywords:** Sepsis, Mitochondria, Respiratory function, Multiple organ failure, Rat muscle, Septic plasma

## Abstract

**Background:**

Although sepsis-induced organ failure is a major cause of death in ICU worldwide, the associated mitochondrial dysfunction is not fully characterized and there is presently no evidence of causality. In this study, we examined whether a central factor in septic plasma could directly affect respiratory function of healthy rat muscle mitochondria.

**Methods:**

ICU patients with severe sepsis or septic shock were recruited within 24 h of admission together with age-matched controls. Blood samples were centrifuged and immediately frozen. Two trials were performed, and mitochondrial respiration was analyzed using an Oxygraph chamber with a Clark-electrode. (1) Isolated mitochondria from the rat skeletal muscle were divided and incubated for 30 min with plasma from patients or postoperative controls (*n* = 10). Respiration was normalized for citrate synthase activity. (2) Permeabilized muscle fibers from rats were divided and incubated with plasma from patients or healthy controls, for 30 and 120 min, and analyzed for mitochondrial respiration (*n* = 10). Respiration was normalized for wet weight. Primary outcome was state 3 respiration, corresponding to the maximal respiration initiated by ADP and energy substrates (malate and pyruvate). *T* test was used for statistical comparison.

**Results:**

No differences in respiratory function of the mitochondria were seen between the groups in either of the experiments. (1) State 3 respiration of isolated mitochondria were 19.9 ± 6.7 vs. 20.2 ± 8.8 nmol O_2_ × U CS^−1^ × min^−1^ for sepsis vs. control, respectively. (2) State 3 respiration for fibers incubated with septic and control plasma were after 30 min 2.6 ± 0.3 vs. 2.4 ± 0.7 and after 120 min 2.5 ± 0.4 vs. 2.5 ± 0.6 nmol O_2_ × mg × w.w^−1^ × min^−1^. Respiratory control ratios were good in all experiments (8.8–11.2), ensuring functioning mitochondria.

**Conclusions:**

These findings indicate that muscle mitochondria are not directly influenced by a factor in plasma of septic patients. The effects seen in mitochondrial function in sepsis may rather be a result of intracellular processes and signaling, such as e.g., production of reactive oxygen species.

## Background

The systemic inflammatory reaction to an infection, sepsis, may progress into severe sepsis or septic shock. In the worst cases, this can develop into multiple organ failure. Both sepsis and, especially, the consequent organ failure remain the major causes of mortality in ICU patients, despite advances in care in the last decades [1]. The cause of the organ failure associated with sepsis in still not fully understood, but metabolic alterations are described [2] and are suggested as a possible mechanism. Mitochondrial dysfunction and derangement are associated with septic shock and are described in different organs in animal models and septic patients in the last decades. However, the methods used to assess mitochondrial function are variable, and the possible mechanism not fully described and causative relationships are not thoroughly established [3–6].

Decreases in mitochondrial protein content, complex I and IV activity, and intramuscular ATP have been described in the skeletal muscle of septic patients with organ failure [7], and low mitochondrial function in the skeletal muscle of septic patients are associated with increased mortality [8]. On the other hand, studies in peripheral blood immune cells (PBMC) show more conflicting results. Increased mitochondrial respiration throughout the course of sepsis in adults, but no difference is seen between survivors and non-survivors [9]. However, Garrabou et al. have shown that oxygen consumption of PBMCs are decreased as compared to healthy controls, but do not state at what time point of sepsis this was assessed [10]. In early pediatric sepsis, PBMCs are exhibiting mitochondrial dysfunction, assessed as decreased spare respiratory capacity and increased uncoupled respiration, as compared to non-septic pediatric ICU patients, and this is normalized at days 5–7 [11].

Isolated mitochondria from the skeletal muscle of a healthy human incubated with plasma from septic patients show a slight, but not significant, decrease in oxygen consumption after 30 min [10]. Also, human umbilical endothelial cells decrease their mitochondrial respiration when incubated with plasma from septic patients compared to controls [12]. These studies suggest a potential circulating factor, directly affecting mitochondrial function in sepsis.

To further investigate whether a central factor, present in plasma of septic patients, could directly affect mitochondrial function, we performed a study on the effect of plasma from human with severe sepsis on healthy rat muscle mitochondria. In a first experiment, we examined how plasma from ICU patients with severe sepsis and septic shock could affect the respiration of isolated mitochondria compared to plasma from age-matched postoperative controls. In a second experiment, we examined the mitochondria in a more physiological situation by incubating permeabilized muscle fibers from the rat muscle with plasma from septic ICU patient or healthy controls.

## Methods

### Study population, ethical considerations, and animal care

For the first part of the study, 10 patients with severe sepsis or septic shock, according to the criteria specified in the 2001 SCCM/ESICM/ACCP/ATS/SIS International Sepsis Definitions Conference [13], were recruited within 24 h of ICU admission and a blood sample was collected together with clinical data. Ten age- (±5 years) and sex-matched controls were recruited from the postoperative care facility after undergoing minor elective surgery. For the second experiment, 10 new ICU patients were recruited, in an identical manner as in the first trial, and 10 healthy age- and sex-matched individuals, without prior surgery, were used as controls. These controls were screened for C-reactive protein (CRP) analysis and by health status questionnaires to rule out ongoing inflammatory process. Patient’s characteristics are presented in Table 1. All blood samples were collected in EDTA tubes, immediately centrifuged at 2000*g* for 10 min to obtain plasma, frozen and stored at −80 °C until experiment.

**Table 1:** Patient characteristics

Subj. no.	Source	Age	Sex	SOFA	Lactate (mmol/L)	Outcome at 30 days
1:1	Urinary tract	79	F	13	3.7	Alive
1:2	Respiratory	55	F	10	4.1	Alive
1:3	Hematogenous	85	M	13	1.2	Dead
1:4	Liver abscess	38	M	9	2.6 (3.2)	Alive
1:5	Soft tissue	40	F	2	1.7	Alive
1:6	Respiratory	51	F	9	1.6	Alive
1:7	Cholangitis/liver abscess	62	M	19	8.8	Dead
1:8	Hematogenous	74	F	3	3.1	Alive
1:9	Unknown	66	M	4	1.1	Alive
1:10	Respiratory	72	M	13	2.8 (3.2)	Alive
2:1	Respiratory	69	F	7	2.3	Alive
2:2	Urinary tract	47	F	1	3.4	Alive
2:3	Abdominal	58	F	11	11.4	Dead
2:4	Abdominal	38	M	12	1.5	Alive
2:5	Unknown	58	M	9	3.0	Alive
2:6	Respiratory	53	M	8	4.1	Alive
2:7	Abdominal	58	M	13	3.6 (6.0)	Dead
2:8	Respiratory	69	F	7	1.9 (2.0)	Alive
2:9	Respiratory	33	M	4	1.5 (2.1)	Alive
2:10	Thoracic abscess	71	F	6	1.3	Alive

Baseline characteristics of ICU patients with severe sepsis. Lactate values are from ICU admission. Numbers in brackets are peak lactate levels if not reached at time of admission. Sequential organ failure assessment (SOFA) score is presented for the day of enrollment in study

### Mitochondrial preparation and respiratory measurements

Male Sprague–Dawley rats (6–8 weeks old, weight 150–250 g) were sacrificed using CO_2_ followed by cervical dislocation. Within 2 min of euthanasia, m. soleus (120–180 mg) were harvested and put in pre-chilled tubes with isolation solution (100 mM sucrose, 100 mM KCl, 50 mM Tris-HCl, 1 mM K_2_HPO_4_, 0.1 mM EGTA, and 0.2 % bovine serum albumin (BSA), pH 7.4) and kept on ice until preparation.

In the first experiment, mitochondria were isolated according as described before [14]. Briefly, muscle specimen was disintegrated, using scissors, in the isolation solution and treated with 1 ml of 0.2 mg/ml protease (Sigma P-4789) for 2 min followed by homogenization (Potter–Elvehjem homogenizer) and washing with isolation solution. The homogenate was centrifuged for 10 min at 4 °C at 750*g*. The obtained supernatant was centrifuged at 4 °C at 10,000*g*. The pellet, containing mitochondria, was washed and suspended in 50 μl of preservation buffer (225 mM mannitol, 75 mM sucrose, 10 mM TRIS-base, 0.1 mM EDTA, and 0.2 % BSA, pH 7.4). The solution was divided into three portions, the one part was kept frozen at −80 °C for later citrate synthase (CS) analysis, and the two portions were incubated with either septic or control plasma and kept on ice in a dark environment for 30 min. After incubation, mitochondrial respiration was analyzed using an Oxygraph chamber (Hansatech DW1; Hansatech, King’s Lynn; Norfolk, UK) at 25 °C in an incubation medium (225 mM mannitol, 75 mM sucrose, 10 mM Tris-base, 10 mM KCl, 10 mM K_2_HPO_4_, and 0.1 mM EDTA, pH 7.0). Seven microliters of 100 mM malate, 3 μl of 10 mM MgCl, and 3 μl 500 mM pyruvate were added before state 3 respiration was started by addition of 7 μl 20 mM ADP. P/O ratios were assessed as the known amount of ADP being phosphorylated divided by oxygen consumed during state 3 respiration.

In the second experiment, the muscle specimen was prepared through a modification of previously described methods [15, 16]. The harvested muscle was stored in a BIOPS solution (2.77 mM Ca K_2_EGTA, 7.23 mM K_2_EGTA, 5.77 mM Na_2_ATP, 6.56 mM MgCl_2_ 6H_2_O, 20 mM taurine, 15 mM Na_2_ phosphocreatine, 20 mM imidazole, 0,5 mM dithiothreitol (DTT), 50 mM MES, pH 7.0). Small pieces (1–3 mg) of muscles were cut and placed in a Petri dish containing BIOPS solution. Fiber bundles were separated under a microscope and then put in a solution containing saponin for 30 min. After this, the fibers are washed in a MiR05 solution (0.5 mM EGTA, 3 mM MgCl_2_, 60 mM K-lactobionate, 20 mM taurine, 10 mM KH_2_PO_4_, 20 mM HEPES, 110 mM sucrose, 1 mM BSA, and pH 7.1) and kept on ice while incubated with plasma from septic patients or healthy controls for 30 and 120 min. One part of the fibers was not incubated and used for control to ensure proper quality of the fibers after the preparation process. Analysis in the Oxygraph was performed in the MiR05 solution with addition of 2 μl 1 M malate and 4 μl 1 M pyruvate, and state 3 respiration was started by addition of 5 μl 160 mM ADP.

### Citrate synthase analysis

Stored mitochondrial suspensions were defrosted and further diluted with a phosphate buffer (pH 7.4) for the CS analyzes. Samples were mixed with a reagent (100 mM Tris, 100 μM DTNB and 50 μM Acetyl CoA, pH 8.0), and 7 mM oxaloacetic acid was used to start the reaction. The change in absorbance, before and after addition of oxaloacetic acid, was measured with a Konelab 20 Analyzer (Thermo electron corporation, USA) at 412 nm and 37 °C.

### Data analysis

All data were blinded for the examiner calculating the respiratory rates. In both experiments, state 3 respiration was approximated as the highest rate of oxygen consumption. In the first experiment, state 4 respiration was derived from the oxygen consumption after depletion of ADP. The data are presented as oxygen consumption per unit of CS to compensate for the varying amount of mitochondria in each experiment. In the second experiment, state 2 respiration was derived from oxygen consumption before addition of ADP. The data are presented per milligrams of wet weight. Student’s *t* test and ANOVA were used for comparison between the paired groups. Data are presented as means ± SD unless otherwise indicated.

## Results

**Fig. 1:**
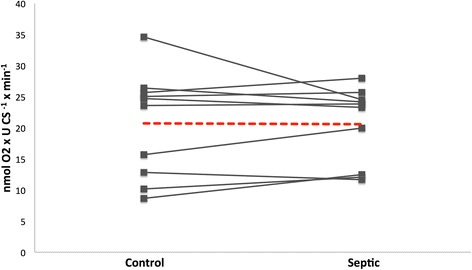
State 3 respiration of isolated rat muscle mitochondria after incubation with plasma. Isolated mitochondria were incubated with either plasma from ICU patients with severe sepsis or postoperative age-matched controls for 30 min. The respiratory rates are expressed per unit of citrate synthase (CS) activity. *Red dotted lines* indicate average values

**Fig. 2:**
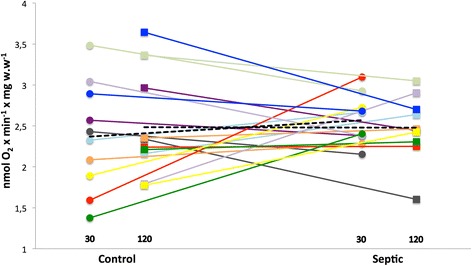
State 3 respiration of permeabilized muscle fibers after incubation with plasma. Fibers were incubated with plasma from ICU patients with severe sepsis or healthy age-matched controls for 30 (*circles*) and 120 min (*squares*). The respiratory rates are expressed per wet weight of the incubated fibers. Each *color* represents a separate patient and its matched control, and *black dotted line* indicates average values

All patients in the study fulfilled criteria for severe sepsis or septic shock with a 30-day mortality of 20 % and initial mean arterial lactate of 3.3 (range 1.1–11.4) mmol/L in both experiments. The quality of the isolated mitochondria and permeabilized muscle fibers were good, as indicated by a respiratory control ratio (RCR) of 8.8–11.2 (Table 2). In the first experiment, no difference was observed between the mitochondria incubated with plasma from septic or postoperative patients (Fig. 1). P/O ratios were 2.22 ± 0.25 for controls and 2.34 ± 0.29 for mitochondria incubated with septic plasma, respectively. In the second experiment, no difference in oxygen consumption could be seen between the fibers incubated with plasma from septic patients or healthy volunteers, neither were there any differences between the different incubation times (Fig. 2).

**Table 2:** Respiratory rates of isolated mitochondria and permeabilized fibers after incubation with plasma

Isolated mitochondria	State 3 (nmol O_2_ × U CS^−1^ × min^−1^)	State 4 (nmol O_2_ × U CS^−1^ × min^−1^)	RCR
Septic 30 min	20.6 ± 6.2	2.1 ± 1.1	11.0 ± 3.5
Post-op 30 min	20.7 ± 8.4	2.0 ± 1.1	11.0 ± 2.4
Permeabilized fibers	State 3 (nmol O_2_ × min^−1^ × mg w.w^−1^)	State 2 (nmol O_2_ × min^−1^ × mg w.w^−1^)	RCR
Control fibers	2.9 ± 0.7	0.27 ± 0.04	11.2 ± 2.3
Healthy 30 min	2.4 ± 0.7	0.25 ± 0.06	9.5 ± 2.0
Septic 30 min	2.6 ± 0.3	0.30 ± 0.07	8.8 ± 1.5
Healthy 120 min	2.5 ± 0.6	0.28 ± 0.13	10.2 ± 3.2
Septic 120 min	2.5 ± 0.4	0.27 ± 0.06	9.5 ± 2.2

Isolated mitochondria and permeabilized fibers from the rat skeletal muscle were incubated with plasma from ICU patients with severe sepsis or matched controls. Respirations in the isolated mitochondria are expressed per citrate synthase (CS) activity and in the muscle fibers per wet weight of the incubated fibers. There were no statistically significant differences between the groups in either experiment

## Discussion

In this study, we examined the effect of plasma from septic patients on rat muscle mitochondrial function. We compared septic plasma to both postoperative and healthy controls for different periods of time and using different modes of incubation (isolated mitochondria as well as permeabilized muscle fibers). All experiments were paired, meaning that each mitochondrial population was incubated with matched controls, to avoid variability in the mitochondrial quality after isolation, and the recruited patients were representative for severe sepsis with a 30-day mortality of 20 %. The good RCR values measured in both parts of the study indicate that the mitochondria were not damaged during the isolation or the permeabilization processes. In neither of the experiments, we observed any difference in mitochondrial respiration between incubations with septic or control plasma.

Our data partly contradicts the tendency towards a decreased respiration of isolated skeletal muscle mitochondria by Garrabou et al. [10]. However, the latter study used slightly different methodology and does not report whether they used paired examinations, which is one of the strengths of our study since a large variation is inherent to the sensitive methods of mitochondrial isolation.

However, there are limitations on the conclusions that can be drawn from the present study. Even though all septic patients are recruited within 24 h from ICU admission, they have received resuscitative treatment and the plasma samples would not necessarily represent the plasma from a native septic process. The plasma levels of catecholamines would probably change during this initial treatment, which would be relevant as a continuous infusion of adrenaline increases respiration in skeletal muscle mitochondria of healthy humans [17]. Most of the patients also received noradrenaline infusion at the time of blood sampling, which has been shown to increase liver mitochondrial respiration, at least in endotoxemic pigs [18]. The plasma composition of the septic patient varies over time [19], and it is possible that we miss some processes facilitated by mediators that are in abundance earlier (or later) in the septic illness.

Our study is also limited to skeletal muscle mitochondria and other organs (e.g. the kidneys and liver) may function differently. This may be why we could not see the decreased respiration that Boulos et al. are describing in endothelial cells incubated with septic serum [12]. Also, a 3-h incubation with septic plasma increased oxygen consumption of healthy white blood cells, however, not as much as healthy plasma [20], and a 12-h incubation of human fibroblast cultures with septic serum decrease O_2_ consumption compared to serum from healthy controls [21]. All these studies use longer incubation times in addition to the use of different human cells. While a prolonged incubation of isolated mitochondria would be interesting to examine, the isolated mitochondria have a limited life span and a longer incubation would be difficult to perform. We tried to address this issue by performing longer incubations of the permeabilized muscle fibers, which are more durable, but without any tendencies towards a change in effect.

Altogether, incubation of tissues and isolated mitochondria has been described in different studies with conflicting results. This may well be due to differences in methodology, but the effect of septic plasma on mitochondrial respiration is still not fully described and may well vary between different tissues or the (patho-)physiological state of the individual tissues.

## Conclusions

The organ failure seen in sepsis often debuts after several days, and if mitochondrial dysfunction is a major factor in that development, we do not think that the present study points towards a primary effect caused by a mediator present in the plasma of septic patients, at least not in the skeletal muscle. It is possible that incubation of intact cells or cultures could give additional knowledge on the mechanisms through which mitochondrial function is affected in sepsis and this would be interesting experiments to sequel our study.
